# Large-scale climatic phenomena drive fluctuations in macroinvertebrate assemblages in lowland tropical streams, Costa Rica: The importance of ENSO events in determining long-term (15y) patterns

**DOI:** 10.1371/journal.pone.0191781

**Published:** 2018-02-08

**Authors:** Pablo E. Gutiérrez-Fonseca, Alonso Ramírez, Catherine M. Pringle

**Affiliations:** 1 Department of Biology, University of Puerto Rico- Rio Piedras, San Juan, Puerto Rico; 2 Department of Environmental Sciences, University of Puerto Rico- Rio Piedras, San Juan, Puerto Rico; 3 Odum School of Ecology, University of Georgia, Athens, Georgia, United States of America; Universitat de Barcelona, UNITED STATES

## Abstract

Understanding how environmental variables influence the distribution and density of organisms over relatively long temporal scales is a central question in ecology given increased climatic variability (e.g., precipitation, ENSO events). The primary goal of our study was to evaluate long-term (15y time span) patterns of climate, as well as environmental parameters in two Neotropical streams in lowland Costa Rica, to assess potential effects on aquatic macroinvertebrates. We also examined the relative effects of an 8y whole-stream P-enrichment experiment on macroinvertebrate assemblages against the backdrop of this long-term study. Climate, environmental variables and macroinvertebrate samples were measured monthly for 7y and then quarterly for an additional 8y in each stream. Temporal patterns in climatic and environmental variables showed high variability over time, without clear inter-annual or intra-annual patterns. Macroinvertebrate richness and abundance decreased with increasing discharge and was positively related to the number of days since the last high discharge event. Findings show that fluctuations in stream physicochemistry and macroinvertebrate assemblage structure are ultimately the result of large-scale climatic phenomena, such as ENSO events, while the 8y P-enrichment did not appear to affect macroinvertebrates. Our study demonstrates that Neotropical lowland streams are highly dynamic and not as stable as is commonly presumed, with high intra- and inter-annual variability in environmental parameters that change the structure and composition of freshwater macroinvertebrate assemblages.

## Introduction

Global climate change is causing organisms to experience new variability in their environments. Freshwater ecosystems are likely to undergo substantial changes, as they closely reflect changes in atmospheric thermal regimes and global hydrological cycles [1, 2]. Long-term studies are key to understanding linkages between stream macroinvertebrates and changes introduced by climatic events (e.g., droughts: [3, 4]), long-term climate cycles (e.g., El Niño: [5, 6]), projected increases in temperature and precipitation variability [7], and impacts resulting from changes in water quality, and physical habitat [8, 9]. In the context of biodiversity research, a long-term dataset is defined as information on the variety and abundance of species (or other taxonomic units) at one or more locations over a considerable period of time [10]. For streams, it can be delimited as at least 5 years, which is the minimum time needed to interpret a meaningful representative range of conditions, such as climate-driven wet and dry cycles or cool and warm years [11]. However, long-term ecological data are scarce [11]. This is a serious challenge in Neotropical regions, where there is only one published long-term ecological study of 15y of duration, linking extreme weather events (e.g., hurricanes and drought), and their effects on decapod populations [12]. Here we examine another long-term (15y) data set to investigate the association between temporal variations in climate (i.e., precipitation and ENSO events) and stream environmental factors, and how they affect macroinvertebrate structure and composition in two lowland streams in Costa Rica.

Tropical streams offer a unique opportunity to understand the effects of climate change, as they experience reduced temporal variation in some environmental characteristics relative to their temperate counterparts. For example, temperature varies less throughout the year, with seasonal changes of only a few degrees, resulting in a more stable metabolic potential relative to high latitude temperate streams [13]. Consequently, Hynes [14] and Jackson and Sweeney [15] suggest that temperature plays a minor role in tropical benthic communities. Other environmental drivers such as precipitation may have higher variability in tropical versus temperate streams. Precipitation is more abundant in many parts of the tropics than at higher latitudes, and can generate strong aseasonal flow regimes [13] that affect aquatic communities (e.g., [16, 17]). Lowland Neotropical streams draining volcanic landscapes can exhibit natural variability in nutrient concentrations (e.g., phosphorus) due to inputs of geothermally-modified groundwater [18]. Nutrients can enhance basal resources (i.e., algae and microbes) that indirectly benefit primary consumers [13]. Neotropical studies along natural nutrient gradients have demonstrated that nutrients play important roles in ecosystem processes, such as leaf litter breakdown [19, 20]. Also, high nutrient concentrations enhance detritus decay rates, microbial activity, and chironomid biomass turnover [21].

Large-scale climate phenomena, such as El Niño Southern Oscillation (ENSO) events, exert important controls over stream ecosystems. A period of ENSO is characterized by complex weather patterns, such as changing wind direction and velocity, temperature and the timing and amount of precipitation over cycles that occur roughly every 3–7 years [22]. Previous studies have found a relationship between the effects of physicochemical variables and large-scale climate phenomena on aquatic organisms. For example, reproductive success of several fish species decreased due to a reduction in the availability of nesting habitat in several streams in Surinam during a severe drought associated with El Niño 1998 [23]. Moreover, a decrease in the abundance and taxonomic richness of aquatic macroinvertebrates due to high water flow was observed during La Niña 1999 in streams draining dry forest in Colombia [24]. Climate change is altering the natural patterns of large-scale climatic phenomena, such as ENSO, causing their range and magnitude to be less predictable [25]. Freshwater ecosystems are vulnerable to climate change and aquatic fauna could be seriously affected due to the reduction in the availability of critical habitats [26, 27, 28]. However, information on the effects of climate change on aquatic ecosystems is limited [28, 29] and understanding how climate change influences aquatic macroinvertebrate assemblages is a challenge and priority for ecologists. To meet this challenge, Durance and Ormerod [30, 31] suggest gathering ecological data temporally extensive enough to determine the interaction between climate change, extreme events and other environmental pressures on streams.

The main objectives of this study were to (i) describe long-term fluctuations in climate and environmental variables, (ii) quantify temporal variation of stream macroinvertebrate assemblages, and (iii) determine which climate and environmental variables most closely reflect observed temporal variations in macroinvertebrates. In addition, one of our study streams was subject to long-term (8y) phosphorus (P) enrichment and we predicted that this would have a positive effect on macroinvertebrate assemblages, as it enhances microbial activity in streams.

## Material and methods

### Study system

La Selva Biological Station (LSBS) (10° 26'N, 84° 01'W) is located on the Caribbean Slope of Costa Rica. LSBS, combined with the Braulio Carrillo National Park, form a biological corridor that covers an elevational range of approximately 2900 m asl to 35 m asl. LSBS has 1563 ha of mature (73% of the reserve) and secondary forest in the wet forest life zone [32], with an annual precipitation range between 4000 to 6000 mm and a bimodal precipitation distribution, with peaks of more than 400mm month^−1^ occurring both from June to July and November to December. The period with least precipitation is from February to April, with March as the driest month [33]. LSBS is drained by a complex network of streams that flow through the forest and are tributaries of two main rivers: Río Puerto Viejo and Río Sarapiquí. Stream water temperature is relatively constant throughout the year and ranges from 24 to 27°C [33].

Streams at LSBS exhibit a wide range of solute concentrations due to natural inputs of solute-rich groundwater, which originates at high elevations where volatile gases from a deep magma source are absorbed into groundwater [34, 35]. This interbasin groundwater flow is cold and enters lowland streams enriched with various chemical constituents, including: P, Ca^2+^, Fe^2+^, Mg^2+^, Na^+^, Si, Cl^−^ and SO_4_^2−^. Geothermally-modified streams are common in volcanically active areas throughout Central America [18]. Seasonal changes in water pH have been observed in some of the streams in LSBS [36]. Previous research described a pH decline during the wet season; in some cases dropping ~3 units between seasons. The magnitude of seasonal pH change is positively related to total precipitation during both the previous dry and wet season. Streams receiving inputs of solute-rich groundwater are better buffered against changes in pH, with solutes such as HCO_3_^-^ acting as a pH buffer [37].

Our study was conducted in Carapa-60 and Saltito-100, two small streams that flow into the Río Sarapiquí. Numbers in the stream name represent the approximate elevation in meters above sea level. We selected a 100 m reach of each stream to sample the different study parameters, so the stream is our sampling unit. Both streams drain mature forest covered by dense riparian vegetation, which provides abundant litter and woody debris to streams. Stream channels are about 1 m wide and 0.25 m deep. Dominant benthic substrata are detritus and sediments (i.e., silt and clay). Stream banks and margins are covered with leaf litter and are partially inundated during high flows, mainly during the wet season.

### Climate data

We used precipitation data from the meteorological station at LSBS (available at (http://www.ots.ac.cr/meteoro/). To examine relationships between macroinvertebrate assemblages at LSBS and global weather patterns, we used the Southern Oscillation Index (here after SOI), which is a measure of the standardized difference in sea level pressure between Darwin (Australia) and Tahiti. The SOI is used as the El Niño Southern Oscillation (ENSO) phase indicator. Thus, the cold La Niña phase of the SOI is characterized by a positive index, whereas the warm El Niño phase is characterized by a negative index. Monthly values of SOI are publicly available through the National Oceanographic and Atmospheric Administration (NOAA; http://www.cpc.ncep.noaa.gov/data/indices/soi).

We related precipitation and discharge using Spearman Rank Correlation (*r*_*s*_) to determine the precipitation necessary to cause a storm and calculate days elapsed since the last storm (DSLS, Carapa-60: *r*_*s*_ = 0.49, *p*<0.001, Saltito-100: *r*_*s*_ = 0.59, *p*<0.001). DSLS has been used as a powerful explanatory variable influencing aquatic macroinvertebrate structure in Neotropical streams [38]. A rain event of >15 mm in 24 h was considered a storm because it doubles discharge at the study streams. This increase of discharge was taken as a disturbance, as headwater streams are easily influenced by small scale differences in local hydrological conditions (e.g., [39, 40, 41]).

### Physicochemical variables

Physicochemical variables were measured parallel with macroinvertebrate sampling: from 1997 to 2004 the macroinvertebrate sampling was monthly, while from 2005 to 2011 it was quarterly. Water pH, temperature, and conductivity were determined in the field using portable multi-probe meters (Hanna Instruments, Woonsocket, RI, USA). Nutrient concentrations were measured by collecting two filtered (0.45-μm Millipore filters) water samples in each stream using new 125 mL bottles. Samples were kept frozen until analyzed at the University of Georgia. NO_3_^−^ N, NH_4_^+^ N, and P (as soluble reactive P: SRP) concentrations were measured using continuous-flow colorimetry and an Alpkem RFA 300 colorimetric analyzer. The Cd reduction, phenate, and ascorbic acid methods were used for NO_3_^−^ N, NH_4_^+^ N, and SRP, respectively [42]. Flow was measured with a Marsh–McBirney current meter, and discharge was estimated using the velocity–area method [43]. We did not measure flow during extreme storm conditions, due to difficulties in accessing the streams during storms, so the values obtained represent discharge during non-extreme storms.

The experimental P enrichment was conducted over 8 y in Carapa-60; P-addition ran from August 1998 to February 2006 [44]. Phosphoric acid (H_3_PO_4_) was added continuously using a Mariotte bottle to increase P concentrations from natural background SRP levels of ~5 to 300 μg L^−1^ SRP, a mean concentration for streams that receive an input of interbasin groundwater inputs at LSBS [34]. SRP was analyzed using the molybdenum blue method [45]. Samples were collected biweekly 10 m downstream of the injection. The whole-stream P enrichment is described in more detail in Ramírez and Pringle [21] and Small *et al*. [44, 46]. Contrary to our study, in which the complete macroinvertebrate assemblage was used as a response variable to the P addition, previous studies focused on the effect of P enrichment on the production of a dominant primary consumer [21] and on the retention of P in the sediments once the P addition was concluded [44, 46].

### Macroinvertebrate sampling

Aquatic macroinvertebrate sampling was conducted monthly from 1997 to 2004 and quarterly from 2005 to 2011. Three core samples (0.006 m^2^ each) were collected per sampling date in each of three different runs along the 100 m study reach where leaf litter was the dominant benthic substrata. These three samples were considered as replicates for each study stream and averaged to represent taxonomic richness and macroinvertebrate abundance per sampling date. The corer was forced into the substrate, cutting through debris, to a depth of ~10 cm. Samples were preserved in formalin (~5%), and macroinvertebrates were later removed from organic material under a dissecting microscope at 40X. Benthic organic matter (BOM) was dried at 70°C for 24 h to determine dry mass and then ashed at 500°C for 1 h to determine ash-free dry mass (AFDM).

Macroinvertebrate samples were identified to the lowest taxonomic level possible (mostly genus, except in Chironomidae, which were identified to subfamily in most cases) using Springer et al. [47]. Biomass was estimated by measuring the length of each individual to the nearest 0.5 mm and applying length–mass relationships [48, more details in S1 File] derived from insects of similar morphology and typically from the same family. Macroinvertebrate abundance and biomass were expressed as ind/m^2^ and g AFDM/m^2^, respectively. We obtained appropriate collecting permits from the National System of Conservation Areas of Costa Rica. Permits are renewed annual and our latest has the number No: 035-2017-ACC-PI.

### Statistical analyses

We tested for the presence of linear trends in environmental factors and macroinvertebrate assemblages with a Mann-Kendall nonparametric trend analysis (M-K), using the *MannKendall* function of the *Kendall* package [49] for R statistical software [50]. The Mann-Kendall analysis works well on small and large data sets; it is not affected by outliers and can be used with irregularly spaced data [51]. Sen’s slope was calculated to determine the magnitude of the trends on the data assessed from Mann-Kendall analysis [52], using the *mannKen* function of the *wq* package [53] for R. Sen’s slope calculates the median of all differences between successive data values to determine the changes per unit time [54].

The relationship between environmental factors influencing macroinvertebrates (i.e., nutrients: SRP, NO_3_−, NH_4_^+^; physicochemistry: pH, conductivity, temperature; hydrological: discharge, DSLS; climatological: mean monthly precipitation, precipitation the day before sampling, SOI; and benthic organic matter) was analyzed with a linear regression model framework, using the *lm* function of the *AICcmodavg* package [55] for R. Variables considered within each model were previously selected with the Artificial Contrast Ensemble (ACE) feature selection method [56], using the *randomForest* package [57]. ACE is a non-linear robust selection method that uses all variables to generate a model and then ranks variables in order of importance. ACE uses these rankings to remove irrelevant predictors (i.e., variables), using random forest classifiers and hypothesis tests that provide *p*-values to assess the statistical significance of an individual predictor.

Multicollinearity among environmental variables within each linear model was assessed with Variance Inflation Factors (VIF), which were calculated with the *vif* function in the *car* package [58] in R. VIF measures how much the variance of the estimated regression coefficients are increased relative to when the predictor variables are not linearly related. Collinearity was determined not to be a problem since all VIF values were less than 10 for all independent variables [59, 60]. Additionally, we used Spearman correlation to detect eventual collinearity for the full set of explanatory variables. All our variables (i.e., physicochemical and climate) were not strongly collinear (|*rs*| <0.60, S1 and S2 Tables). ACE and VIF results are shown in the S3 Table. Violation of standard statistical assumptions was checked by visually examining residuals and Q-Q plots for all reported models.

Model selection approach based on Akaike’s Information Criteria corrected for small sample sizes (AICc; [61]) was used to rank models in terms of their capacity to explain the relationship among environmental variables and macroinvertebrate structure and composition. The single best supported model was selected on the basis of the AIC weight, calculated to evaluate the relative likelihood of a model [62]. Models that were considered poor as compared to the best one, were ranked using Delta AIC (best fit model Delta AIC = 0); Delta AIC quantifies how strongly models compete [61]. When multiple models were supported (Delta AIC <2), we used model averaging to increase precision of inference [61, 55]. AIC has the advantage of comparing several competing models and selecting the most parsimonious model that still provides an adequate fit to the data. All the data was first standardized ([x-μ]/σ) to reduce variability due to differences in variable units, while keeping the ratio (i.e., variation) within each variable. AIC was run with R.

We used non-metric multidimensional scaling (nMDS) based on Bray-Curtis coefficient as a measure of monthly dissimilarities in macroinvertebrate assemblage abundance or presence/absence. Each ordination was run with 50 random starting configurations. Cluster analysis was used to identify composition similarity and visualize groups in the nMDS. Subsequently, we tested significance of the observed groups using analysis of similarities (ANOSIM). ANOSIM scores range between 0 and 1, with higher values corresponding to greater dissimilarity between groups. ANOSIM used Bray-Curtis distance and 9999 permutations. Finally, the similarity percentage–species contributions (SIMPER) procedure [63] was used to identify which taxa were responsible for dissimilarities among groups in nMDS and supported by the ANOSIM (Bray-Curtis distance).

Permutational multivariate analysis of variation (PERMANOVA) was applied to assess differences in environmental variables among groups formed in nMDS and supported by ANOSIM. Bray–Curtis dissimilarity was the chosen distance measure to perform PERMANOVA; 9999 randomizations permutations were used. nMDS was run with PC-ORD Software (version 5, [64]).

## Results

### Long-term patterns and trends of environmental variables

**Fig 1:**
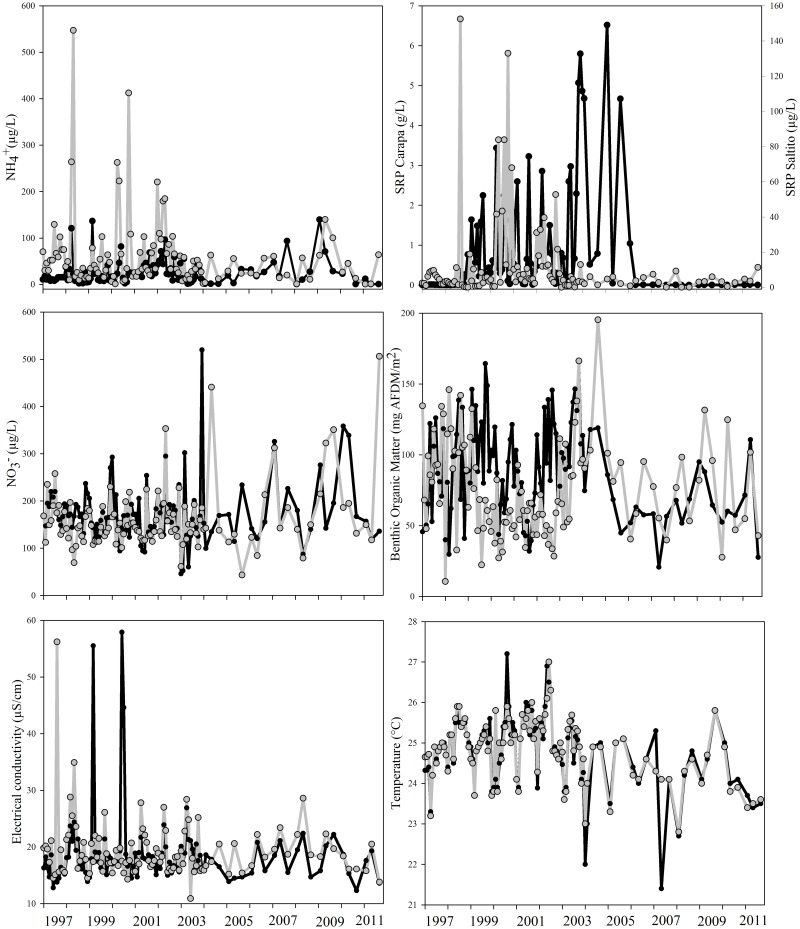
Monthly temporal patterns of environmental variables between 1997 to 2011 in Carapa-60 (Black line) and Saltito-100 (Gray line).

**Fig 2:**
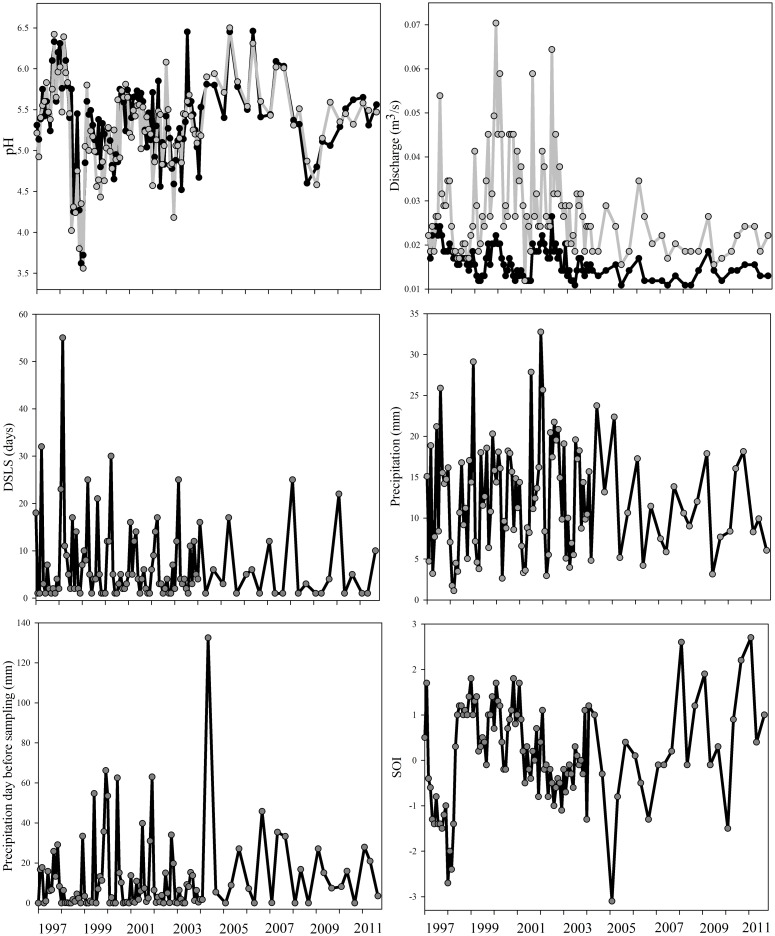
Monthly temporal patterns of climate and environmental variables between 1997 to 2011 in Carapa-60 (Black line) and Saltito-100 (Gray line). Last four graphs show overall patterns for both streams.

The long-term monthly record of environmental variables showed high temporal variability in both streams (Figs 1 and 2). Most variables fluctuated without showing a significant trend of increasing or decreasing over time (i.e., NH_4_^+^, NO_3_^−^, BOM, conductivity, pH, precipitation, DSLS, and SOI; all *p*>0.05). We found significant temporal trends for a few variables. SRP was experimentally manipulated in Carapa-60, where it ranged from ~5 μg L^−1^ to values as high as 6541.9 μg L^−1^ during the P-addition. Monthly SRP concentration in Saltito-100 showed no significant decreasing trend over the study (*p*>0.05) and ranged from ~0.1 μg L^−1^ to 152.45 μg L^−1^. Temperature decreased by ~1°C throughout the study and was significant for Carapa-60 (M-K = -0.13, *p* = 0.04, Sen’s slope = -0.04), ranging from 21.4 to 27.2 °C, and in Saltito-100 (M-K = -0.14, *p* = 0.03, Sen’s slope = -0.05) ranging from 22.8 to 27.0 °C. Average monthly discharge ranged from 0.011 m^3^s^−1^ to 0.027 m^3^s^−1^ in Carapa-60, and from 0.011 m^3^s^−1^ to 0.070 m^3^s^−1^ in Saltito-100, which showed consistently higher mean discharge than Carapa-60. Over time, significant decreases in discharge were observed in Carapa-60 (M-K = -0.35, *p*< 0.0001, Sen’s slope = -0.0005) and Saltito-100 (M-K = -0.15, *p* = 0.02, Sen’s slope = -0.0004).

### Long-term patterns of macroinvertebrate assemblages

A total of 99 taxa (mostly genera) belonging to 47 families of 9 orders of aquatic macroinvertebrates were collected in Carapa-60 and Saltito-100 during the 15-y study. The most diverse order was Diptera with 19 taxa in Carapa-60 and 17 in Saltito-100. Odonata and Trichoptera were the second and third most diverse orders. Diptera was also the most abundant order, with more than 89% of the total abundance for each stream. Chironomidae represented approximately 80% of the total abundance of macroinvertebrates, and 90% of the total abundance of Diptera. Contributions from non-insects to the total abundance were modest, with approximately 3.14% and 3.63% of the total abundance in Carapa-60 and Saltito-100, respectively. Contributions from other orders of insects to total abundance were minor (<3.05%). Biomass in Carapa-60 was dominated by Diptera (44.57%), Odonata (32.87%) and Trichoptera (15.42%); the rest of macroinvertebrates represented 7.15% of total biomass. In contrast, biomass in Saltito-100 was dominated by Odonata (55.40%); Diptera (26.13%) and Trichoptera (8.88%); the rest of macroinvertebrates represented 9.59% of the total biomass.

**Fig 3:**
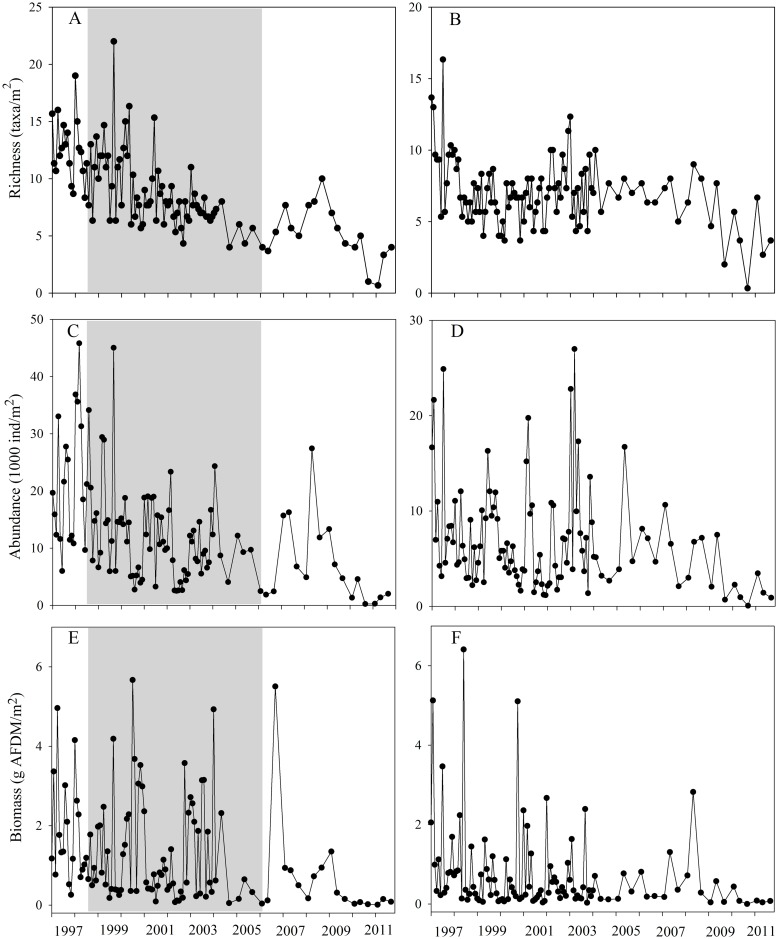
Long term fluctuations in taxonomic richness, abundance and biomass in Carapa-60 (A, C, E) and Saltito-100 (B, D, F). Gray area shows 8y of P-enrichment in Carapa-60.

Taxonomic richness in Carapa-60 was highest at the beginning of the study, decreasing significantly over time (M-K = -0.56, *p*< 0.0001, Sen’s slope = -0.70). The maximum number of taxa was found in September 1999 (22 taxa/m^2^); while the minimum number was found in February 2011 (0.67 taxa/m^2^, Fig 3A). In Saltito-100, richness was stable at the beginning of the study, but it also showed a significant decrease towards the end (M-K = -0.14, *p* = 0.03, Sen’s Slope = -0.14). The maximum number of taxa sampled was 16.33 taxa/m^2^ in July 1997; while the minimum was 0.33 taxa/m^2^ in September 2010 (Fig 3B).

Monthly macroinvertebrate abundance varied widely in both streams throughout the study. Carapa-60 showed a strong decrease with time (M-K = -0.36, *p*< 0.0001, Sen’s slope = -1.09). Maximum abundance was 46,583.6 ind/m^2^ in March 1998; while the minimum was 209.6 ind/m^2^ in September 2010 (Fig 3C). Abundance in Saltito-100 showed a significant decrease over the study (M-K = -0.19, *p* = 0.003, Sen’s slope = -0.29). Maximum abundance was 27,143.2 ind/m^2^ in March 2003, while the minimum was 52.4 ind/m^2^ in September 2010 (Fig 3D).

Likewise, annual macroinvertebrate biomass fluctuated throughout the study in both streams. High biomass occurred early and decreased at the end. Macroinvertebrate biomass in Carapa-60 decreased (M-K = -0.27, *p*< 0.0001, Sen’s slope = -0.074) and fluctuated between 5.666 g AFDM/m^2^ in July 2000 to 0.00691 g AFDM/m^2^ in February 2011 (Fig 3E). Biomass in Saltito-100 also decreased (M-K = -0.21, *p*< 0.001, Sen’s slope = -0.026) and fluctuated between 6.408 g AFDM/m^2^ in June 1998 to 0.00026 g AFDM/m^2^ in September 2010 (Fig 3F).

### Relationships between macroinvertebrate assemblages and physicochemical parameters

Model selection criteria showed that of all measured parameters, hydrological and climatic variables had the greatest potential to explain temporal patterns in macroinvertebrate composition. In Carapa-60, taxonomic richness was best described by a model that included discharge, DSLS, and SOI (Table 1). Discharge and DSLS were positively related to richness, while SOI was negatively related. The best model identified by AIC for macroinvertebrate abundance in Carapa-60 included DSLS and average precipitation. DSLS showed a positive relationship with abundance, while the average precipitation was negatively related. No model of the variables were able to describe macroinvertebrate biomass (Table 1). Richness in Saltito-100 was best described by BOM and SOI. Organic matter was positively related to richness, while SOI was negatively related. Although average precipitation was the best model that described macroinvertebrate abundance, AIC analysis results showed no support for this simple model. The model showed a negative relationship between average precipitation and abundance. No model of the variables explained macroinvertebrate biomass variability (Table 1).

**Table 1:** Akaike Information Criterion (AICc) model results for the composition and structure of macroinvertebrate assemblages. Model were ranked using Delta AIC. The column “Signal” indicates the direction, (+) positive and (-) negative, of the effect. The column “Model Parameters” indicates if the effects are (*) multiplicative or (+) additive. K, number of parameters in the model; AICc Wt, AIC weight; Cum. Wt, Cumulative AIC weight.

Stream	Response Variable	Signal	Model Parameters	K	AICc	Delta AIC	AICc Wt	Cum. Wt
Carapa-60	Taxonomic Richness	Discharge+ DSLS+ SOI-	Discharge * DSLS * SOI	4	299.43	0.00	0.48	0.48
		Discharge+ SOI-	Discharge + SOI	5	300.80	1.37	0.24	0.73
		Discharge+ DSLS+ SOI-	Discharge + DSLS + SOI	5	301.13	1.70	0.21	0.93
		DSLS+ SOI-	DSLS * SOI	9	305.30	5.87	0.03	0.96
		Discharge+ DSLS+	Discharge * DSLS	3	306.85	7.42	0.01	0.97
		Discharge+ DSLS+	Discharge + DSLS	4	307.21	7.78	0.01	0.98
		Discharge+	Discharge	3	307.96	8.53	0.01	0.99
		Discharge+ SOI-	Discharge * SOI	5	308.25	8.82	0.01	0.99
	Abundance	DSLS+ Average Precipitation-	DSLS * Average Precipitation	5	285.49	0.00	0.28	0.28
		DSLS+ SOI-	DSLS * SOI	5	286.17	0.68	0.20	0.48
		Average Precipitation- SOI-	Average Precipitation + SOI	4	286.26	0.77	0.19	0.67
		Average Precipitation- SOI-	Average Precipitation * SOI	5	287.17	1.68	0.12	0.8
		DSLS+ SOI-	DSLS + SOI	4	287.67	2.18	0.09	0.89
		Average Precipitation -	Average Precipitation	3	287.96	2.47	0.08	0.97
	Biomass		None entered the model					
Saltito-100	Taxonomic Richness	Benthic organic Matter+ SOI-	Benthic organic Matter * SOI	4	300.60	0.00	0.53	0.53
		Benthic organic Matter+ SOI-	Benthic organic Matter + SOI-	3	302.17	1.58	0.24	0.77
	Abundance		Intercept	3	297.40	0.00	1.00	1.00
		Average Precipitation-	Average Precipitation	2	312.44	15.04	0.00	1.00
	Biomass		None entered in the model					

### Temporal patterns of macroinvertebrate assemblages

**Fig 4:**
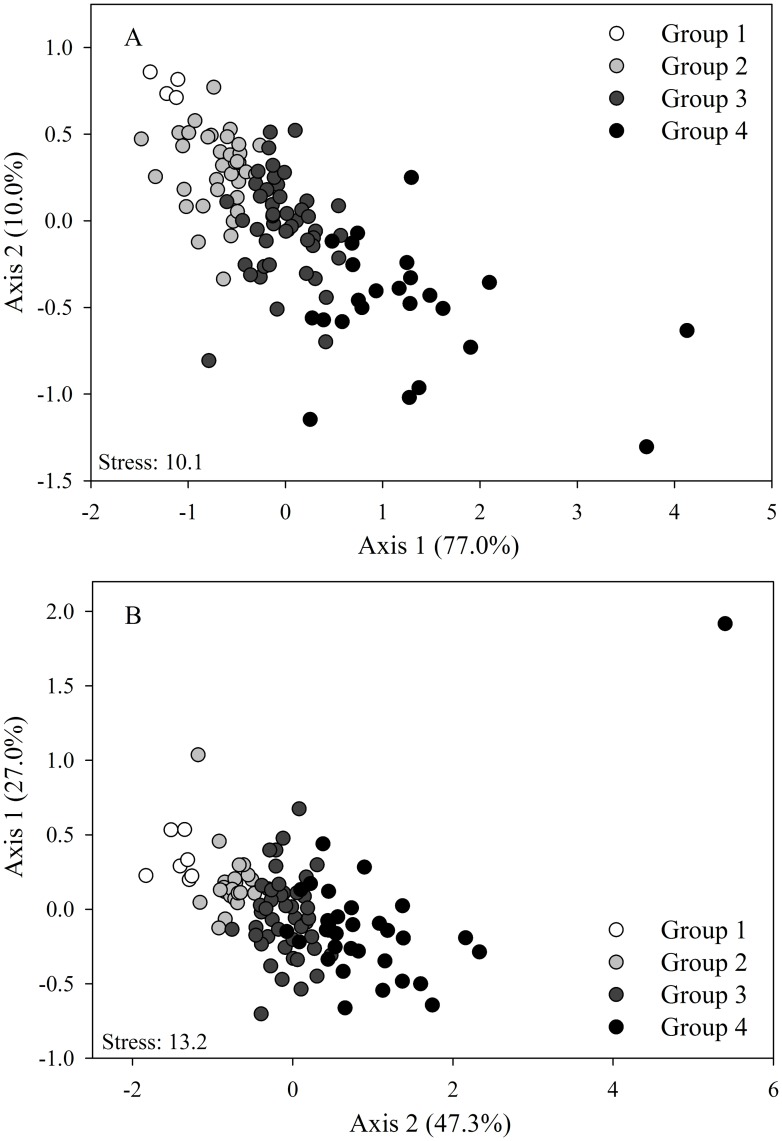
non-Metric multidimensional scaling ordinations (nMDS plots) on the basis of the Bray-Curtis dissimilarity measure of abundance in (A) Carapa-60 and (B) Saltito-100.

Ordination analysis of macroinvertebrates based on abundance and presence/absence suggested the formation of groups in the data set. We used cluster analysis to delineate major groups (clusters not shown) and mark them in nMDS plots; the observed groups were ordered from highest to lowest abundance, starting with group 1. The nMDS of macroinvertebrate abundance in Carapa-60 showed four well defined groups (Fig 4A). Likewise, ANOSIM analysis confirmed that assemblage abundance differed statistically among these four groups with a high dissimilarity value (R = 0.61, *p* = 0.0001, Table 2). SIMPER analysis showed that three Chironomidae taxa accounted for more than 60% of assemblage dissimilarity among groups. Based on the cluster analysis, nMDS of macroinvertebrate abundance in Saltito-100 also resulted in four groups (Fig 4B). ANOSIM confirmed that assemblage abundance differed significantly among these groups (R = 0.60, *p* = 0.0001, Table 2). SIMPER analysis revealed that three taxa of Chironomidae and Ceratopogonidae accounted for more than 60% of assemblage dissimilarity among groups.

**Table 2:** ANOSIM analysis results among groups formed in nMDS plots.

Stream		Groups compared	R	*p*
Carapa-60				
	Presence	Group 1 vs Group 2	0.57	0.0001
		Group 1 vs Group 3	0.94	0.0001
		Group 2 vs Group 3	0.46	0.0001
	Abundance	Group 1 vs Group 2	0.62	0.0001
		Group 1 vs Group 3	0.92	0.0001
		Group 1 vs Group 4	0.82	0.0001
		Group 2 vs Group 3	0.48	0.0001
		Group 2 vs Group 4	0.88	0.0001
		Group 3 vs Group 4	0.57	0.0002
Saltito-100				
	Presence	Group 1 vs Group 2	0.24	0.0001
	Abundance	Group 1 vs Group 2	0.50	0.0001
		Group 1 vs Group 3	0.96	0.0001
		Group 1 vs Group 4	0.87	0.0001
		Group 2 vs Group 3	0.49	0.0001
		Group 2 vs Group 4	0.77	0.0001
		Group 3 vs Group 4	0.48	0.0001

**Fig 5:**
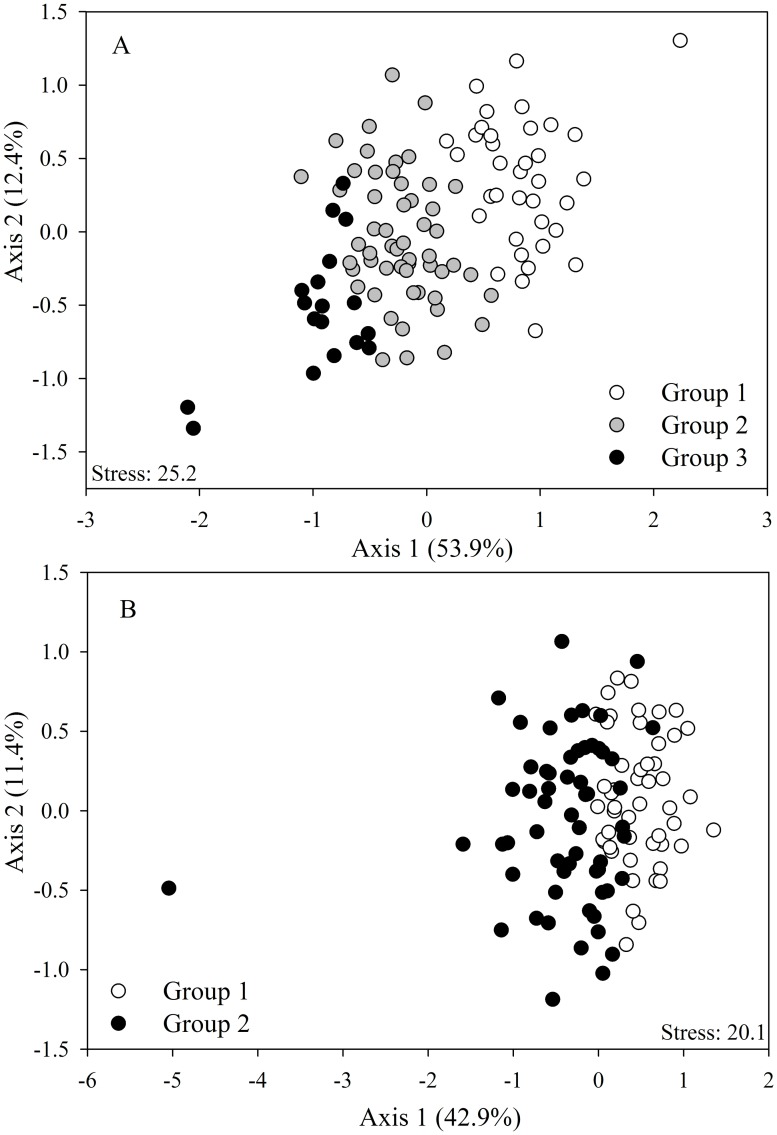
non-Metric multidimensional scaling ordinations (nMDS plots) on the basis of the Bray-Curtis dissimilarity measure of presence/absence of macroinvertebrates in (A) Carapa-60 and (B) Saltito-100.

Cluster analysis of macroinvertebrates based on presence/absence of macroinvertebrates also showed clearly defined groups (clusters not shown); the observed groups were ordered from highest to lowest presence of organisms, starting with group 1. The three groups delineated for Carapa-60 are shown in the nMDS plot (Fig 5A), and ANOSIM indicated a strong separation among them (R = 0.61, p = 0.0001, Table 2). SIMPER analyses revealed that the taxa with highest contribution to the global dissimilarity (i.e., >50%) were: Diptera (*Hexatoma*, Orthocladinae, *Xestochironomus*, Tipulidae, and *Chrysops*), Ephemeroptera (*Caenis*, *Farrodes*, and *Americabaetis*), Coleoptera (*Hexacylloepus*), Trichoptera (*Macronema* and *Phylloicus*), Odonata (*Epigomphus*) and non-insects (Oligochaeta, Polychaeta and Nematoda). In Saltito-100, cluster analysis identified two groups, but nMDS showed a weak segregation between these groups, implying that assemblage structure among months was more similar (Fig 5B). The low strength of ANOSIM values supports the weak segregation (R = 0.24, *p* = 0.0001, Table 2). SIMPER analyses showed that Diptera (Orthocladinae, *Hexatoma*, *Xestochironomus*, and Tipulidae), Ephemeroptera (*Farrodes*), Coleoptera (*Hexacylloepus*), Trichoptera (*Macronema* and *Phylloicus*), Odonata (*Epigomphus*) and non-insects (Oligochaeta, Acari, Copepoda and Nematoda) accounted for more than 50% of assemblage dissimilarity among groups.

### Relationship between groups and main environmental variables

PERMANOVA detected that differences among nMDS groups were associated with the same major physicochemical variables identified in the AIC analysis. In the AIC analysis discharge, DSLS, and SOI were included in the best model that described the presence of macroinvertebrates in Carapa-60. In contrast, PERMANOVA indicated that only discharge explained differences among groups (F = 6.16, *p* = 0.003, Table 3). Discharge was higher for group 1 and decreased slightly until lowest for group 3 (Table 4). For Saltito-100, PERMANOVA identified significant differences in benthic organic matter (F = 6.80, *p* = 0.01, Table 3) and SOI (F = 12.97, *p*<0.001, Table 3) among nMDS groups based on presence/absence. Organic matter was higher for group 1 compared with the group 2. Group 1 was characterized by a mean SOI that indicated dry months, while mean SOI in group 2 tended to positive value associated with high precipitation (Table 4).

**Table 3:** PERMANOVA analysis results of groups formed using ANOSIM analysis and the relationships with the main environmental variables.

Stream		Groups compared	Discharge		SOI		DSLS		Average Precipitation		Organic Matter	
			F	*p*	F	*p*	F	*p*	F	*p*	F	*p*
Carapa-60												
	Presence	Group 1 vs Group 2	12.89	**<0.001**	1.31	0.256	2.10	0.158	--	--	--	--
		Group 1 vs Group 3	4.52	**0.035**	0.075	0.777	2.07	0.158	--	--	--	--
		Group 2 vs Group 3	0.02	0.881	0.44	0.502	0.73	0.397	--	--	--	--
	Abundance	Group 1 vs Group 2	--	--	--	--	16.92	**0.003**	5.42	**0.024**	--	--
		Group 1 vs Group 3	--	--	--	--	18.90	**0.002**	5.70	**0.019**	--	--
		Group 1 vs Group 4	--	--	--	--	27.47	**<0.001**	12.80	**<0.001**	--	--
		Group 2 vs Group 3	--	--	--	--	<0.1	0.989	0.29	0.584	--	--
		Group 2 vs Group 4	--	--	--	--	2.59	0.112	8.84	**0.005**	--	--
		Group 3 vs Group 4	--	--	--	--	2.75	0.098	6.25	**0.013**	--	--
Saltito-100												
	Presence	Group 1 vs Group 2	--	--	12.97	**<0.001**	--	--	--	--	6.80	**0.01**
	Abundance	Group 1 vs Group 2	--	--	--	--	--	--	3.30	**0.078**	--	--
		Group 1 vs Group 3	--	--	--	--	--	--	8.91	**0.004**	--	--
		Group 1 vs Group 4	--	--	--	--	--	--	10.85	**0.003**	--	--
		Group 2 vs Group 3	--	--	--	--	--	--	3.91	**0.054**	--	--
		Group 2 vs Group 4	--	--	--	--	--	--	6.58	**0.014**	--	--
		Group 3 vs Group 4	--	--	--	--	--	--	0.89	0.347	--	--

**Table 4:** Mean and standard deviation of the main environmental variables identified by AIC for each group.

Stream			Discharge		SOI		DSLS		Average Precipitation		Organic Matter	
			Mean	SD	Mean	SD	Mean	SD	Mean	SD	Mean	SD
Carapa-60												
	Presence	Group 1	0.018	0.004	0.00	1.28	9.03	11.74	--	--	--	--
		Group 2	0.015	0.001	0.27	0.95	6.28	6.16	--	--	--	--
		Group 3	0.015	0.004	0.09	1.07	4.89	5.82	--	--	--	--
	Abundance	Group 1	--	--	--	--	14.25	19.62	2.70	3.66	--	--
		Group 2	--	--	--	--	5.55	7.44	4.89	5.89	--	--
		Group 3	--	--	--	--	5.89	7.40	5.11	6.42	--	--
		Group 4	--	--	--	--	2.94	4.55	5.19	6.39	--	--
Saltito-100												
	Presence	Group 1	--	--	-0.24	0.99	--	--	--	--	84.55	38.02
		Group 2	--	--	0.48	1.06	--	--	--	--	67.93	28.39
	Abundance	Group 1	--	--	--	--	--	--	6.15	2.69	--	--
		Group 2	--	--	--	--	--	--	9.89	5.54	--	--
		Group 3	--	--	--	--	--	--	13.01	6.37	--	--
		Group 4	--	--	--	--	--	--	14.44	6.93	--	--

For nMDS groups based on abundance, PERMANOVA showed that in Carapa-60 significant differences were related to DSLS (F = 10.12, p<0.001, Table 3) and average precipitation (F = 5.59, p = 0.001, Table 3). Months from group 1 were those with the most days elapsed since the last major storm, and the number of days decreased consecutively until reaching group 4. Also, months in group 1 were characterized by having less rainfall, which increased until reaching group 4 with the most precipitation (Table 4). Nonetheless, average precipitation was not supported as a best model in AIC in Saltito-100 composition; PERMANOVA analysis showed significant differences in precipitation among groups (F = 5.19, *p* = 0.002), with group 1 having low precipitation and increasing consecutively until the months of group 4 (Table 3).

## Discussion

Our long-term data set demonstrates that lowland Neotropical streams are dynamic environments that lack intra-annual periodicity and have large inter-annual variability. Contrary to the supposition of temporal stability or seasonality for streams draining Neotropical humid forests (e.g., [65, 66, 67]), headwater streams at LSBS in Costa Rica are highly variable environments. We consistently observed that macroinvertebrate assemblages fluctuated over time in our two study streams in response to environmental variables that were strongly related to the hydrologic regime (e.g., rainfall, discharge). Even fluctuations in macroinvertebrates in the experimentally P-enriched stream were found to be associated with hydrology rather than nutrients. The lack of interaction between nutrients and macroinvertebrate assemblages was unexpected. We predicted that enhanced nutrient levels would stimulate macroinvertebrate assemblages. While previous studies at LSBS have shown significant effects on basal trophic levels and processes, (i.e., fungal biomass, microbial respiration), effects on invertebrate consumers may be subtle [20, 68]. Moreover, we found evidence that fluctuations in stream physicochemistry and insect assemblage structure are ultimately the result of large-scale climatic phenomena, as suggested by the SOI (i.e., via changes in hydrology). The SOI is an indicator of the intensity of El Niño / La Niña events, which are important regulators of climate and influence temperature and precipitation patterns across the globe [69, 70].

ENSO events play a key role in modifying environmental conditions and, therefore, the dynamics of many populations and ecosystems. Previous studies have found supporting evidence during recent ENSO events, especially strong ones during the 1980s and 1990s. In African savannas, annual changes in ungulate populations have been related to rainfall deficits, which affect plant growth during severe ENSO years [71, 72]. Meanwhile, in marine ecosystems, drastic changes in temperature and salinity associated with both La Niña and El Niño were related with high variability in zooplankton abundance, as well as an abnormal spawning of the tropical bivalve *Donax dentifer* in the coasts of South America [73, 74]. In terrestrial ecosystems, regional shifts in the abundance of three raptor species have been associated with SOI temperature responses, as their movement followed favorable local conditions for breeding [75]. Magnusson *et al*. [76] found a positive correlation between rodent density and SOI due to the increase in local rain and consequent decrease of fire extent in the Amazonian savanna; fire and rainfall lead to higher than average seedling growth, favoring increases in rodent abundance. Lastly, drought during the 1997–1998 ENSO episode was associated with widespread burning that affected 40,000 km^2^ of standing Amazonian forest [77]. Effects have been also described for LSBS, such as Clark *et al*. [78], Silva *et al*. [79]; thus, it is not surprising that the SOI is associated with macroinvertebrate variability in the two protected and small lowland forest streams of this study. At LSBS, El Niño years are often characterized by drier than average dry seasons, with severe drought and exceptionally high temperatures, which have been related to decreases in liana density and diversity (e.g., [80]).

Our previous studies have shown that ENSO conditions are associated with deviations in the patterns of nutrient chemistry in streams of LSBS. Long-term monitoring of SRP concentrations in streams revealed an increase in the annual mean SRP fluxes, responding to the strong ENSOs of 1992 and 1998–1999 [81]. Changes in mean annual SRP fluxes from the Salto watershed increased from 7.3 kg/ha year in 1997 to 16 kg/ha year in 1998–1999 (ENSO), and then fell to 2.5 kg/ha year in 2001 (post ENSO). Triska *et al*. [81] suggested that this large increase in SRP export was caused by a low flow induced by ENSO, which gradually releases SRP from the sediments into pore water and riparian groundwater. Likewise, a severe pH decline in poorly-buffered streams of LSBS was observed after an extended drought, including the drought followed by ENSO 1998–1999. Intense drought could contribute to increased labile soil carbon, thus promoting high levels of dissolved CO_2_ and a drop below 4.0 pH in low-buffered streams [36, 38]. For the 1998–1999 ENSO period, which was designated among the top five El Niño rankings since 1871 by Wolter and Timlin [82], Ramírez *et al*. [38] found a decrease in insect abundance and biomass in several streams of LSBS. Nevertheless, even though our long-term data set recorded this incident, we did not observe an important assemblage reduction during this event in comparison to the reduction we observed at the end of this study, which could be associated with a combination of hydrological and climatic factors. Although little is known about the effects of El Niño on streams at LSBS, much less is known about the effects of La Niña. La Niña is related to an increase in precipitation, which leads to periods of high discharge that negatively impact organism composition and structure (e.g., [80]). Our results showed a decrease in macroinvertebrate richness and abundance at the end of the study, which coincides with more positive and severe SOI episodes (i.e., more common La Niña events).

Precipitation and associated changes in discharge influence the structure and composition of macroinvertebrate assemblages in different environments [5, 83, 84]. Periods of low and moderate precipitation result in high macroinvertebrate abundance, while high precipitation may shift assemblages to low abundances (e.g., [17]). Discharge can also influence the relative success of different species [85, 86]. High discharge plays key roles in driving the structure and composition of benthic assemblages [87, 88], as the drag force of flowing water can cause catastrophic downstream drift of individuals [89]. Our study shows that macroinvertebrate richness and abundance decreased with increasing discharge and was positively related to the number of days elapsed since the last strong event (see Table 1). McMullen and Lytle [88] demonstrated a significant negative effect of floods on the overall density of macroinvertebrates within the first days of a flood, as well as an increase in the number of macroinvertebrates within the first 10 days after a flood.

Benthic organic matter is a major energy component for stream food webs, and it is also a refuge for aquatic macroinvertebrates [90, 91]. Benthic organic matter can fluctuate in response to discharge and leaf litter inputs from riparian areas [92]. Previous studies (e.g., [93]) have shown a positive relationship between aquatic macroinvertebrates and organic matter; an increase in debris caused an increase of dominant detritivores, such as Chironomidae. We found that some taxonomic groups, such as Chironomidae, contribute greatly to overall assemblage dynamics, which could be favored by the benthic organic matter that is dominant in the sampled habitat. Similar results have been found by Feio *et al*. [83] in streams of Portugal, where Chironomidae dominated assemblages. Changes in the macroinvertebrates assemblages of three regions in northern California were mainly caused by changes in the abundance of chironomids [5].

Nutrients were not major factors related to macroinvertebrate assemblage dynamics, even given our long-term phosphorus addition. As expected, we observed low nutrient concentrations in our two study streams (with the exception of the experimental P-enrichment period in Carapa-60), which are typical in streams without inputs of interbasin groundwater in LSBS [20, 38]. Moreover, previous studies have demonstrated that P effects in stream ecosystems at La Selva are more evident at basal trophic levels. Hence, fungal biomass and leaf breakdown [20] and microbial respiration rates [68] are all positively enhanced by artificial P-amendments and naturally high P levels. More rapid organic matter decay rates have both been related to an increase in microorganism and fungal activity associated with nutrient-rich systems. Rosemond *et al*. [20] observed a positive relationship between leaf decay rates and fungal and invertebrate biomass across naturally heterogeneous P landscapes; but these factors were not enhanced in our artificially P-enriched stream. Exclusion of macroinvertebrates showed that P, instead of macroinvertebrates, contributed largely to leaf decay, possibly improving microbial processes. Also, Ramírez *et al*. [68] found that microbial respiration rates on *Ficus* and mixed leaves were positively related to a natural P gradient in streams at LSBS; also, an experimental P-enrichment in Carapa produced an increase in microbial respiration similar to high-P streams. High microbial respiration, fungal biomass and invertebrate density were higher in enriched and naturally high-P streams than in low-P streams, when leaf litter from three different species with different qualities (concentrations of cellulose, lignin and tannins) were compared [94]. Nitrogen does not appear to be limiting, as was demonstrated by Stallcup *et al*. [95], who found no effect of N addition on leaf breakdown, microbial respiration, ergosterol and leaf chemistry in both the P-enriched and the reference streams.

Nutrient effect in our study streams appear to be highly variable. We did not observe an effect of SRP or any other nutrient on macroinvertebrate abundance, biomass or secondary production, despite the eight-year P addition in Carapa-60. While insect growth and excretion rates have been found to be more responsive to nutrients, responses are variable (e.g., [21, 46]). Ramírez and Pringle [21] found differences in daily growth rates and annual biomass turnover rates of chironomids associated with phosphorus levels, including a faster growth rate of small larval Chironomidae in Carapa during enrichment. Small *et al*. [46] found that the P demands of chironomids differed across streams with a contrasting nutrient input. Hence, chironomids from high P streams grew faster with an increase in litter P content, while the growth rates of chironomids from low P streams did not respond to a dietary increase in P. Growth rate did not change in chironomids of Carapa-60 (P enrichment stream), but the P-excretion rate did increase. It is surprising that even after eight years of P addition, chironomids of this low P stream were not able to benefit from the extra P to produce new biomass. Small *et al*. [46] suggested that local genetic adaptation among chironomid populations could potentially be responsible for differences in P demands, and argue that microevolution could explain this lack of differences in Carapa-60.

Other environmental variables that we measured did not appear to be drivers of macroinvertebrate dynamics. At LSBS, stream conductivity reflects the heterogeneous locations of geothermal seepages, ranging from 22–440 μScm^-1^ [96]; so conductivity is commonly used to identify the limit of interbasin groundwater flow. Temporal variation of conductivity in both streams was rather small and no long-term patterns were observed. Water temperature data show a decrease in stream water temperature, which was unexpected since air temperature increased over 22y in LSBS. Mohseni and Stefan [97] demonstrated that air/stream temperature relationships are not always consistent, fluctuating between a flat to an S-shaped relationship according to the temperature range, travel time (i.e., time it takes for a particle to travel between two arbitrary points) and geology. The annual decrease in average stream temperature might have a negative impact on some ecological processes such as leaf litter decomposition, which is stimulated by greater microbial activity in high tropical temperatures (e.g., [98]). Furthermore, temperature variability was consistent within the range described in previous studies in LSBS [38, 96]. Stream pH showed a wide variation in streams of LSBS [36]. Moreover, long-term studies in streams with a wide variety of groundwater inputs have showed a seasonal trend, with pH increasing during the dry season and decreasing through the wet season [36]. Stream pH has been shown to be important in controlling macroinvertebrate assemblages in streams at LSBS; for example, Ramírez *et al*. [38] showed a decrease in insect density and biomass in six streams in LSBS due to an episodic acidification event in 1998. However, in this study, we did not observe a strong effect of temperature or pH on assemblages. It is possible that other large scale factors (e.g., precipitation, SOI) may be masking their influence.

### Implications and future challenges

Climate change model scenarios predict an increase in extreme weather events, which includes a spatiotemporal redistribution of precipitation patterns resulting in more severe flood and drought periods through 2100 [99]. Our long-term analysis shows high variability in precipitation and a clear trend in discharge, with a directional reduction in both streams over the study period. Consistent with our results, long-term meteorological data of rainfall in LSBS has shown a reduction in the annual mean precipitation from 1963 to 2014 (see meteorological data form LSBS, http://www.ots.ac.cr/meteoro/). Furthermore, regional studies for Central America using the Regional Climate Change Index predict a reduction in precipitation and an increase in precipitation variability in the future scenarios, identifying Central America as a “hotspot” and one of the most sensitive and vulnerable tropical areas susceptible to climate change [100, 101]. Additionally, empirical long-term data for Central America have shown nonsignificant trends in precipitation showing increasing or decreasing values for several stations across the region; this results in a lack of a coherent signal and specific predictions [102]. Even though the incidences of very high precipitation events have increased in the region, which could lead to an abrupt reduction of macroinvertebrate richness and abundance [103], Rauscher *et al*. [104] predict a decrease in summer precipitation and an intensification of the midsummer drought for the next 100 years in Central America. Although there is high uncertainty in future climatic scenarios for the tropics, our study indicates that conditions that lead to increases in stream discharge or floods will likely result in reductions in macroinvertebrate richness and abundance.

Moreover, climate change will probably intensify ENSO activity due to increased greenhouse gases, which cause warming in the ocean surface and affect atmospheric circulation patterns [105, 106, 107]. Recent studies have shown an increase in the intensity of El Niño, indicating it has almost doubled in the past three decades [108]. Furthermore, Yeh *et al*. [109] has suggested that El Niño events occur much more frequently under the projected global warming scenarios, while future extreme La Niña events are expected nearly double their frequency [107]. Interannual variations of Central American climate will be less predictable, since their climate is directly influenced by the surrounding oceans [110]. Steinhoff *et al*. [111] projected variations of the ENSO predictions for Central America and northwest South America using a Coupled Model Intercomparison Project with 15 Global Climate Models for the complete period of 2006–2100. While results vary greatly among the models considered, the authors found support for increases in ENSO frequency associated with greater greenhouse gas concentrations in future climate; this consensus was not found for the duration or amplitude of ENSO events. Steinhoff *et al*. [111] suggest that an increase in ENSO frequency corresponds to an increase in the potential for extreme rainfall in Central America. Given the strong relation between ENSO events and stream dynamics, further efforts to improve ENSO predictions will also help us understand how tropical streams will be affected by future climate.

In summary, this study documents the effects of large scale climate (i.e., ENSO, precipitation) and hydrological variables (i.e., discharge) on the structure and composition of macroinvertebrates in two lowland tropical streams in Costa Rica. While our results are based solely on the study of two streams inside a protected forest, this is the most extensive and consistent data set ever collected in a Neotropical freshwater ecosystem. Long-term studies, such as this one, are essential for observing realistic effects on macroinvertebrate structure and composition and to better understand stream dynamics in the face of climate change.

## Supporting information

S1 File Equation details of length-dry mass relationship [48] used in this study. (DOCX)

S1 Table Spearman’s rank correlation coefficients for variables included in the Carapa-60 models (see Materials and methods for descriptions of variables). (DOCX)

S2 Table Spearman’s rank correlation coefficients for variables included in the Saltito-100 models (see Materials and methods for descriptions of variables). Correlations for climatic variables are shown in S1 Table and were used in the analyses of both streams.
(DOCX)

S3 Table Results of variable selection using Artificial Contrast Ensemble (ACE, with *randomForest* function), model significance, and multicollinearity test using Variance Inflation Factors (VIF, with *vif* function). (DOCX)
