# Supplementary material for: Large-scale climatic phenomena drive fluctuations in macroinvertebrate assemblages in lowland tropical streams, Costa Rica: The importance of ENSO events in determining long-term (15y) patterns
Source: PLoS One. 2018 Feb 8;13(2):e0191781. doi: 10.1371/journal.pone.0191781 (PMC5805265; doi:10.1371/journal.pone.0191781)
Supplement: S1 File — (DOCX) [file pone.0191781.s001.docx]

**Supporting information.**

**S1 File. Equation details of length-dry mass relationship [48] used in this study.**

Biomass of aquatic organisms is widely used to determine secondary productivity, seasonality, life history, and transfer of energy throughout food webs in freshwater ecosystems. Weighing organisms directly can be used to obtain their biomass. However, this process can be tedious and result in the loss of organisms. Also, erroneous calculations can be made due to the preservative and the preservation time of the organisms.

Alternatively, length-dry mass relationships have been used to obtain organism biomass. Indirect estimation of dry mass from the linear relationship between body size and biomass is more efficient, compared to direct determination of individual mass. The relationship between body size and dry mass is usually described as a power function:

DM= *a* L*^b^*

where DM is dry mass (mg), L is body length (mm) and *a* and *b* are fitted regression constants. Additionally, to transform this measurement to wet mass, the most used correction factor is 0.9 for ethanol.
