# Supplementary material for: Large-scale climatic phenomena drive fluctuations in macroinvertebrate assemblages in lowland tropical streams, Costa Rica: The importance of ENSO events in determining long-term (15y) patterns
Source: PLoS One. 2018 Feb 8;13(2):e0191781. doi: 10.1371/journal.pone.0191781 (PMC5805265; doi:10.1371/journal.pone.0191781)
Supplement: S1 Table — (DOCX) [file pone.0191781.s002.docx]

**Supporting information.**

**S1 Table.** **Spearman’s rank correlation coefficients for variables included in the Carapa-60 models (see Materials and Methods for descriptions of variables).**

|  | **SRP** | **NH_4_^+^** | **NO_3­_^-^** | **OM** | **pH** | **Temp.** | **Cond.** | **Disch.** | **DSLS** | **Precip. Day** | **Precip.** |
| --- | --- | --- | --- | --- | --- | --- | --- | --- | --- | --- | --- |
| **SRP** |  |  |  |  |  |  |  |  |  |  |  |
| **NH_4_^+^** | -0.01 |  |  |  |  |  |  |  |  |  |  |
| **NO_3­_^-^** | -0.12 | 0.01 |  |  |  |  |  |  |  |  |  |
| **OM** | 0.33 | -0.17 | 0.09 |  |  |  |  |  |  |  |  |
| **pH** | -0.14 | 0.00 | -0.10 | -0.19 |  |  |  |  |  |  |  |
| **Temp.** | 0.13 | 0.14 | -0.01 | 0.06 | -0.00 |  |  |  |  |  |  |
| **Cond.** | 0.05 | 0.18 | -0.31 | -0.00 | 0.10 | 0.18 |  |  |  |  |  |
| **Disch.** | -0.00 | -0.02 | 0.45 | 0.11 | -0.14 | 0.09 | -0.34 |  |  |  |  |
| **DSLS** | 0.21 | -0.11 | -0.12 | 0.03 | 0.09 | -0.00 | -0.14 | -0.18 |  |  |  |
| **Precip. Day** | -0.13 | 0.03 | 0.09 | -0.01 | 0.06 | -0.03 | -0.05 | 0.17 | -0.59 |  |  |
| **Precip.** | 0.15 | -0.13 | 0.36 | 0.19 | -0.16 | 0.11 | -0.44 | 0.49 | -0.47 | 0.28 |  |
| **SOI** | 0.10 | -0.14 | -0.00 | 0.11 | -0.21 | -0.03 | -0.03 | -0.17 | 0.05 | -0.11 | -0.03 |
