# Supplementary material for: Large-scale climatic phenomena drive fluctuations in macroinvertebrate assemblages in lowland tropical streams, Costa Rica: The importance of ENSO events in determining long-term (15y) patterns
Source: PLoS One. 2018 Feb 8;13(2):e0191781. doi: 10.1371/journal.pone.0191781 (PMC5805265; doi:10.1371/journal.pone.0191781)
Supplement: S2 Table — Correlations for climatic variables are shown in S1 Table and were used in the analyses of both streams. (DOCX) [file pone.0191781.s003.docx]

**Supporting information.**

**S2 Table.** **Spearman’s rank correlation coefficients for variables included in the Saltito-100 models (see Materials and Methods for descriptions of variables). Correlations for climatic variables are shown in S1 Table and were used in the analyses of both streams.**

|  | **SRP** | **NH_4_^+^** | **NO_3­_^-^** | **OM** | **pH** | **Temp.** | **Cond.** | **Disch.** |
| --- | --- | --- | --- | --- | --- | --- | --- | --- |
| **SRP** |  |  |  |  |  |  |  |  |
| **NH_4_^+^** | 0.34 |  |  |  |  |  |  |  |
| **NO_3­_^-^** | 0.22 | 0.18 |  |  |  |  |  |  |
| **OM** | -0.17 | 0.02 | -0.12 |  |  |  |  |  |
| **pH** | 0.00 | 0.12 | -0.05 | 0.15 |  |  |  |  |
| **Temp.** | 0.21 | 0.21 | -0.01 | 0.10 | -0.10 |  |  |  |
| **Cond.** | -0.12 | 0.13 | -0.22 | 0.11 | 0.07 | 0.10 |  |  |
| **Disch.** | 0.17 | 0.05 | 0.26 | -0.43 | -0.16 | 0.11 | -0.41 |  |
| **DSLS** | 0.05 | -0.04 | -0.13 | 0.15 | -0.06 | 0.02 | 0.19 | -0.16 |
| **Precip. Day** | -0.14 | 0.00 | 0.15 | -0.14 | -0.01 | -0.06 | -0.13 | 0.16 |
| **Precip.** | -0.04 | -0.06 | 0.32 | -0.27 | -0.12 | 0.10 | -0.43 | 0.59 |
| **SOI** | -0.01 | -0.19 | -0.00 | -0.06 | -0.41 | -0.03 | -0.11 | -0.04 |
