# Supplementary material for: Large-scale climatic phenomena drive fluctuations in macroinvertebrate assemblages in lowland tropical streams, Costa Rica: The importance of ENSO events in determining long-term (15y) patterns
Source: PLoS One. 2018 Feb 8;13(2):e0191781. doi: 10.1371/journal.pone.0191781 (PMC5805265; doi:10.1371/journal.pone.0191781)
Supplement: S3 Table — (DOCX) [file pone.0191781.s004.docx]

**Supporting information.**

**S3 Table.** **Results of variable selection using Artificial Contrast Ensemble (ACE, with *randomForest* function), model significance, and multicollinearity test using Variance Inflation Factors (VIF, with *vif* function).**

| **Stream** | **Response variable** | **Model** | **Variable** | **ACE** | **Model significance** | | **VIF** |
| --- | --- | --- | --- | --- | --- | --- | --- |
|  |  |  |  | ***p*-value** | **F** | ***p*-value** |  |
| Carapa-60 | Taxonomic richness | Discharge * DSLS * SOI | -- | -- | 3.36 | 0.002 | -- |
|  |  |  | Discharge | <0.001 | -- | -- | 1.22 |
|  |  |  | DSLS | <0.001 | -- | -- | 1.61 |
|  |  |  | SOI | <0.001 | -- | -- | 1.23 |
|  | Abundance | DSLS * Average Precipitation | -- | -- | 12.26 | <0.001 | -- |
|  |  |  | DSLS | <0.001 | -- | -- | 3.82 |
|  |  |  | Average Precipitation | <0.001 | -- | -- | 1.60 |
| Saltito-100 | Taxonomic richness | Benthic organic Matter+ SOI | -- | -- | 5.744 | 0.001 | -- |
|  |  |  | Benthic organic Matter | <0.001 | -- | -- | 1.01 |
|  |  |  | SOI | <0.001 | -- | -- | 1.01 |
|  |  | Average Precipitation | -- | -- | 18.24 | <0.001 | -- |
|  |  |  | Average Precipitation | 0.001 | -- | -- | -- |
